# Climate, housing, energy and Indigenous health: a call to action

**DOI:** 10.5694/mja2.51610

**Published:** 2022-06-16

**Authors:** Simon Quilty, Norman Frank Jupurrurla, Ross S Bailie, Russell L Gruen

**Affiliations:** ^1^ Australian National University Canberra ACT; ^2^ Julalikari Council Aboriginal Corporation Tennant Creek NT; ^3^ University Centre for Rural Health Lismore NSW

**Keywords:** Poverty, Climate change

The convergence of excessive heat, poor housing, energy insecurity and chronic disease has reached critical levels

Most Australians take safe housing and uninterrupted electricity for granted. Yet in remote Indigenous communities, low quality poorly insulated housing and energy instability are common.^1^ Most houses require prepaid power cards, resources are meagre, financial literacy is low, and people often have to choose between power and food. New evidence reveals extreme rates of prepaid electricity meters’ disconnection in these communities,^2^ making people with chronic diseases who depend on cool storage and electrical equipment particularly vulnerable. The convergence of excessive heat, poor housing, energy insecurity and chronic disease has reached critical levels in many parts of northern Australia, and a multisectoral response is needed to avert catastrophe. Medical professionals have a key role to play.

The Northern Territory, for example, is experiencing extreme heat stress (Box 1). The summer of 2019–20 was 4°C above the long term average, and the town of Katherine, which previously averaged 6 days per year over 40°C, had 56 such days in 2019.^3^ The year before, Tennant Creek recorded 28 days above 40°C in one month,^4^ and Alice Springs recorded its hottest day since records began.^5^

Over recent summers it’s been too hot. Particularly them hot days when the power do go off, we all get out of the house, we always sit outside. I normally just sit under the sprinkler or under the hose, over my head.

Everything’s been dying out here around Tennant Creek. All the water in the rock holes went dry. The heat killed animals. Even the spinifex went black, it looked like it’d been burnt or poisoned. A lot of them trees around town, not them native trees but cedar trees and African mahogany, all them mango trees around Tennant Creek, all died, nothing left. That heat would just come too low, the heat wave killed the whole lot. (Norman Frank Jupurrurla, Warramungu Elder and dialysis patient from Tennant Creek)

Box 1 **Unprecedented summer extremes for (A) 2018–2019 and (B) 2019–2020**

**Figure:**
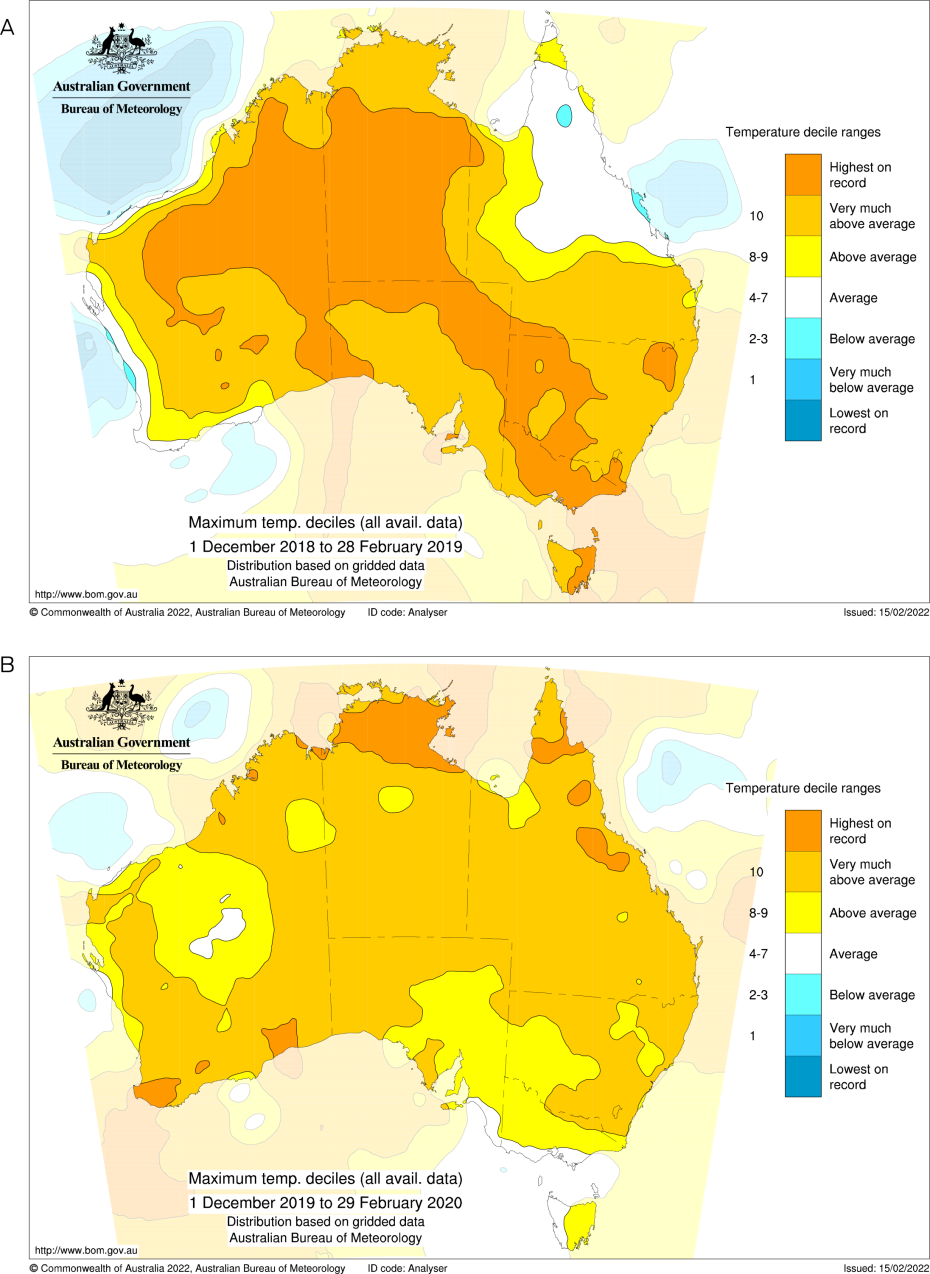
Source: Bureau of Meteorology; reproduced with authorisation from Pandora Hope.

Around the world it has been shown that chronic disease and heat stress combine to exacerbate morbidity and mortality.^6^, ^7^ The rate of chronic diseases in remote communities is high, and many people depend on heat‐sensitive medications such as insulin. Most medications have recommended storage temperatures below 30°C, and yet for many this may be impossible to achieve.

Some people on the outskirts of Tennant Creek still live in old tin houses and there’s no running water, there’s no power, there’s not even a toilet, not even an old drop toilet. Kids go to school from there and people go to work … “You’ll end up getting cooked in that tin house today,” that’s what we say … There’s a renal patient out there, living in a camp, he got renal at the same time as me and the renal bus go out there, pick him up in the camp, near his tin shed, take him to dialysis with me.

The association between health and housing quality in remote communities is well documented, as is “health hardware”, such as a functioning refrigerator, necessary for the practices of healthy living.^8^ It has been two decades since the last major survey of Indigenous housing quality, when it was shown that more than half of remote households did not have a refrigerator.^9^ It is not known whether this has changed. Even less well understood is the impact that energy poverty has on the function of such hardware. For people experiencing energy poverty, refrigeration for food and medication, air conditioning, power for oxygen concentrators, continuous positive airway pressure machines, home dialysis equipment and so on become critical concerns. Health care providers need to be alert to the implications for clinical practice.

Doctors should start asking the question, if you’ve got a fridge or not. I reckon that’s what these doctors think, every Wumpurrarni [Indigenous person] lives the same as a whitefella and they’ve got everything the same. But not all of us got a fridge. When doctors put people on insulin and educate them, when dieticians talk to them and tell them, “You need to be on insulin”, they don’t ask that question “Do you have a fridge? Where do you stay? What kind of condition you live in?”

When the power disconnects because we run out of money [on a prepaid meter], you have to hurry up. If you catch it in a few hours, you’ll be lucky, but if I’m out somewhere on the weekend and it goes off, everything goes off in the fridge. When I come in late or at night and find that the power’s been off, everything’s off in the fridge, so I’ve had to throw everything out.

Current building codes provide little protection for residents against environmental harms. The NT building code legislation has two tiers, with more rigorous requirements in urban than rural areas. However, remote houses are outside of these two tiers and building contractors are not even required to be registered with the Building Practitioners Board.^10^ As a result, many dwellings, particularly older ones, lack the basics — appropriate passive cooling design, structural integrity, insulation in ceiling cavities — and poor quality housing fiercely exacerbates energy poverty.^1^ Particularly in areas of Australia where temperature extremes now extend for months, houses turn into heat caves, much like a car parked in the hot sun. Even if air conditioning is available, cooling poorly designed and uninsulated houses is far more expensive than it is for better quality dwellings. Because of the much higher energy requirements, just when the power is needed to keep people safe from extreme heat, it most often disconnects.^2^

I’m in a brick house. I’m in an old brick house still and it’s really hot in that house … It’s really hot in summertime. When you’ve got winter, that brick is really cold. In winter it’s the other way around, that house of mine.

Beyond building codes, remote communities face many other structural and institutional barriers to addressing energy poverty and thermal safety. For example, the Department of Housing and Community Development mandates mechanical air conditioning for houses in the arid region, but only ceiling fans in the hotter and more humid tropical region.^11^ Furthermore, remote Indigenous communities do not benefit from energy security protections afforded the majority of Australians under the National Energy Retail Rules, which, among other things, protects customers facing financial hardship.^12^ Remote Indigenous communities are also locked out of solutions to energy poverty such as rooftop solar photovoltaic systems installations.^13^ There is only one Indigenous public house in the NT with a rooftop solar system installed, and negotiations around feed‐in tariffs and utility support for such solutions have been lacking (Box 2).

Box 2 **Installation of the first rooftop solar system on a public house in a remote Indigenous community or town camp, which at the time of publication remains unconnected 3 months later**

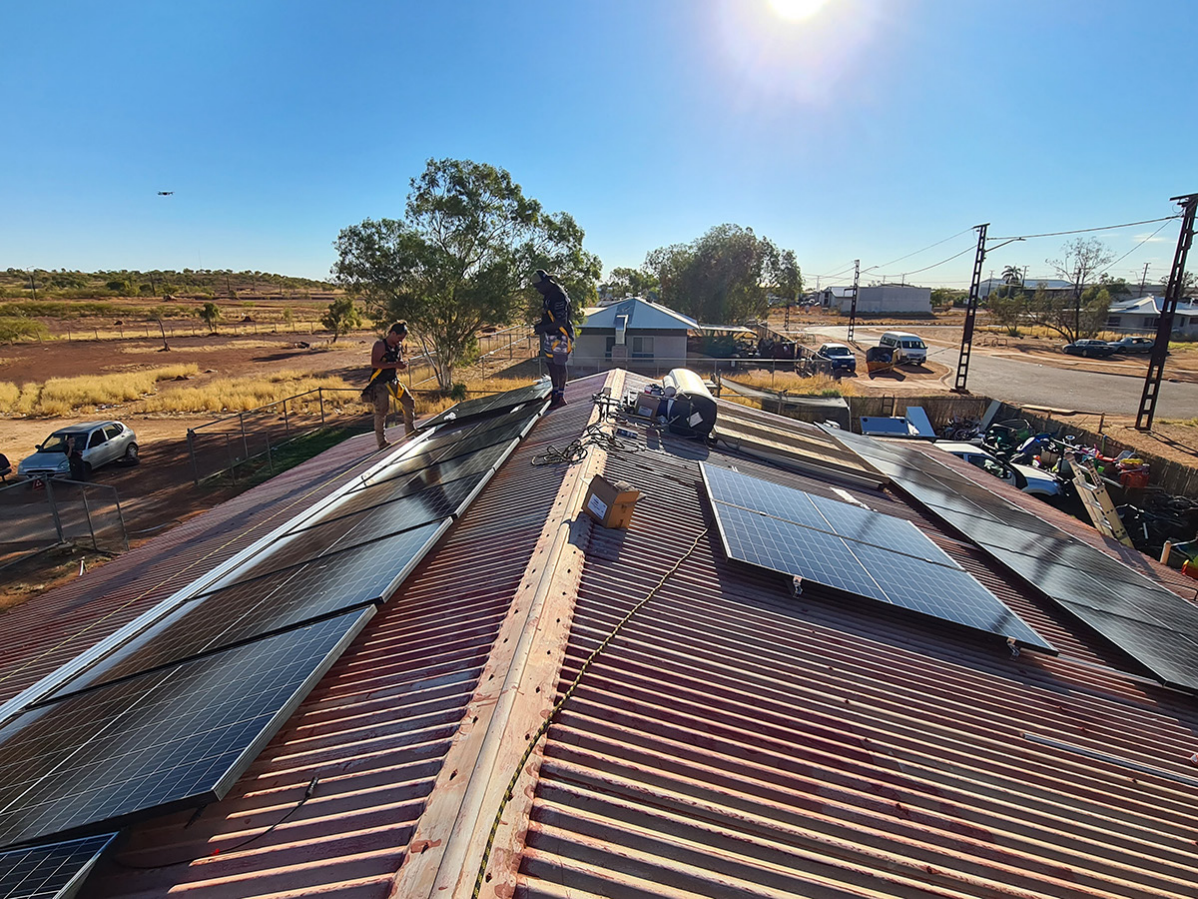

## The role of the medical profession

Health care practitioners need to be cognisant of the direct impacts of heat on their patients’ health and recognise comorbid conditions and risk factors that increase vulnerability to heat.^7^ It is equally important for clinicians to understand people’s access to thermal safety, capacity to appropriately store medications, and resources to power essential health infrastructure such as oxygen concentrators. Explicit inquiry about housing conditions, the availability of refrigeration and air conditioning, and how regularly the power turns off may be particularly revealing.

Beyond individual patient care, the medical profession can engage with pharmaceutical and health care device industries to ensure that details of thermal stability of products are available to clinicians. For example, although almost all pharmaceuticals’ labelling mandate storage below 30°C, it is likely that many products can withstand higher temperatures. On the other hand, some antibiotics, antidiabetic medications, antiepileptics and warfarin, which are all medications regularly prescribed in Indigenous communities, are known to degrade in the heat.^14^ In the NT, clinicians need to understand the thermal stability of everything they prescribe.

There are also many unknowns and misconceptions about how to protect human health from extreme heat, for instance, air conditioning and cooling technology may not be a panacea.^15^ Therefore, a high priority is to develop an evidence‐based agenda of heat adaptation and health, including public health responses to extreme heat events, that provides a robust basis for advocacy and action as we all try to adapt to a rapidly heating world.

In the context of heat, housing, energy, and chronic disease, there is much that health professionals can advocate for to reduce structural inequities that perpetuate Indigenous peoples’ health risks and relative disadvantage.^16^ This begins with health professions bearing witness to current housing disparities and their impact on health and safety of remote community residents. The profession can highlight the association of housing quality, heat stress and energy security in relation to demand on health services so it is given appropriate priority in government decision making.

In relationship to housing and health, our profession needs to advocate for strengthening of building codes and housing standards for remote Indigenous dwellings (Box 3). Identifying and rectifying deteriorating infrastructure, reviewing maintenance standards to ensure dwellings are fit for purpose into the future, and ensuring appropriate design and quality construction of new buildings is all of urgent priority in a warming climate. This includes enhanced responsiveness of public utilities in the interest of the health and safety of remote community residents.

Box 3 **Remote Indigenous housing mandate**

**Table:** 

• Remote community housing solutions need to be enshrined in community control. • Every house should be thermally safe. • Every house needs a refrigerator that has uninterrupted electricity supply. • Existing housing infrastructure needs to be reviewed or retrofitted to ensure tenants are provided with dwellings of appropriate thermal design and construction. • Remote communities need the same legislative protections for housing and associated utilities as mainstream Australia. • Residents with chronic health conditions need culturally appropriate Housing‐for‐Health Liaison Officers who can assist with housing induction, negotiate housing repairs or rectification between agencies or trades, quality inspection, basic energy audits, and rectification processes of a broad range of everyday housing challenges with a “no‐wrong‐doors” approach.^17^ • Health care professionals need to have clear processes with electricity providers to ensure secure and uninterrupted energy supply for people with relevant health vulnerabilities. • Remote Indigenous communities should be economic beneficiaries of renewable energy technology.

You’ve got to stand strong. If you’re going to give up on them and stop holding them accountable, they’ll give up on you too and won’t do what they are supposed to, that’s how they are. If you stop making noise, they’ll just sit there quietly and do nothing, they wouldn’t worry and would leave things broken as they are. They don’t give a damn about you. The way I see it I’ve been in my house for nearly 5 years, and I’ve been trying to get help with housing and providers coming round, trying to ask them for help or support or fix plumbing. I’ve had to report it over and over and over before they do anything about it. If I give up, they’ll give up. But I am not ever going to give up.

And finally, Indigenous leadership at all levels, from community to state to federal, is fundamental to ensuring that housing solutions are designed and controlled by the people who will call these dwellings home. Health professionals are well positioned to advocate for Indigenous‐led multisectoral approaches to comprehensively address the need for healthy and safe living conditions in a warming climate.

The community needs to be in charge of what they want done in their housing and how they want their lifestyle, and be allowed to make the solutions. Then they can bring that to the table, to the housing and to the providers. Then it’s not coming from some government from Canberra, it’s not coming from some politician. It’s coming from us, it’s coming straight from the horse’s mouth and straight from the ground, from the grassroots, that’s where you’ve got to listen, from their home.

## Open access

Open access publishing facilitated by Australian National University, as part of the Wiley ‐ Australian National University agreement via the Council of Australian University Librarians.

## Competing interests

No relevant disclosures.

## Provenance

Not commissioned; externally peer reviewed.
